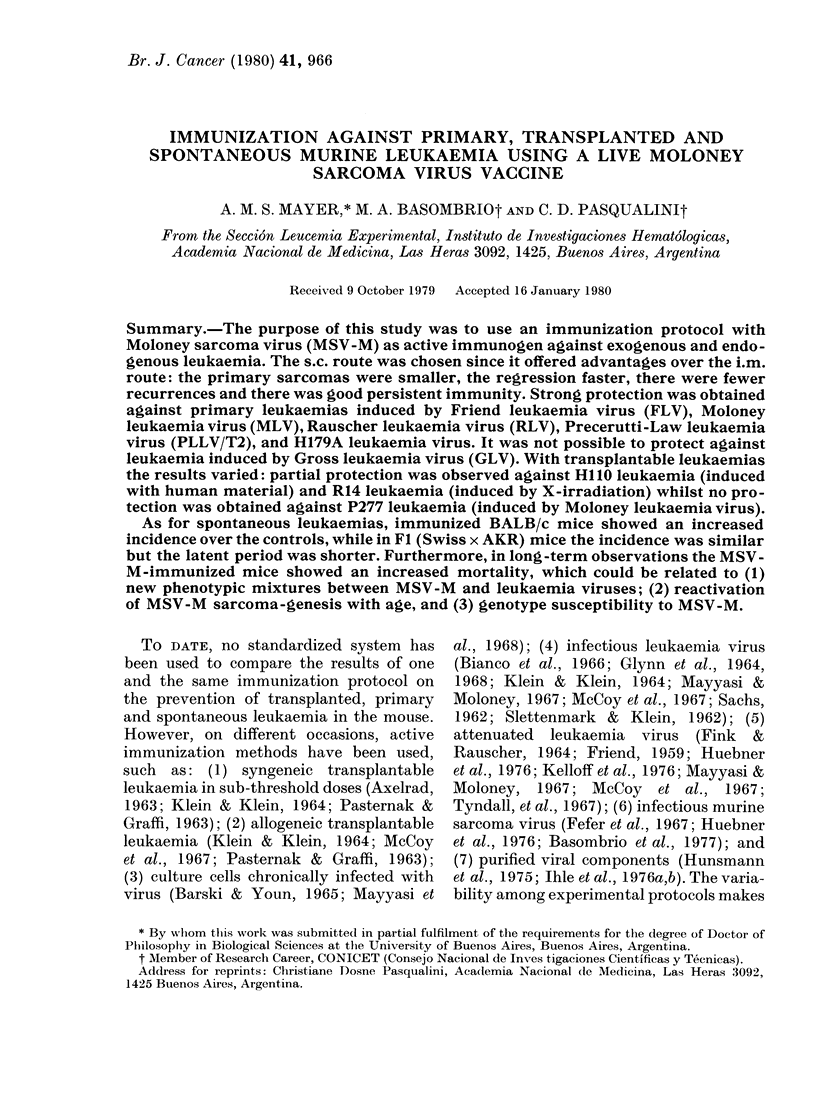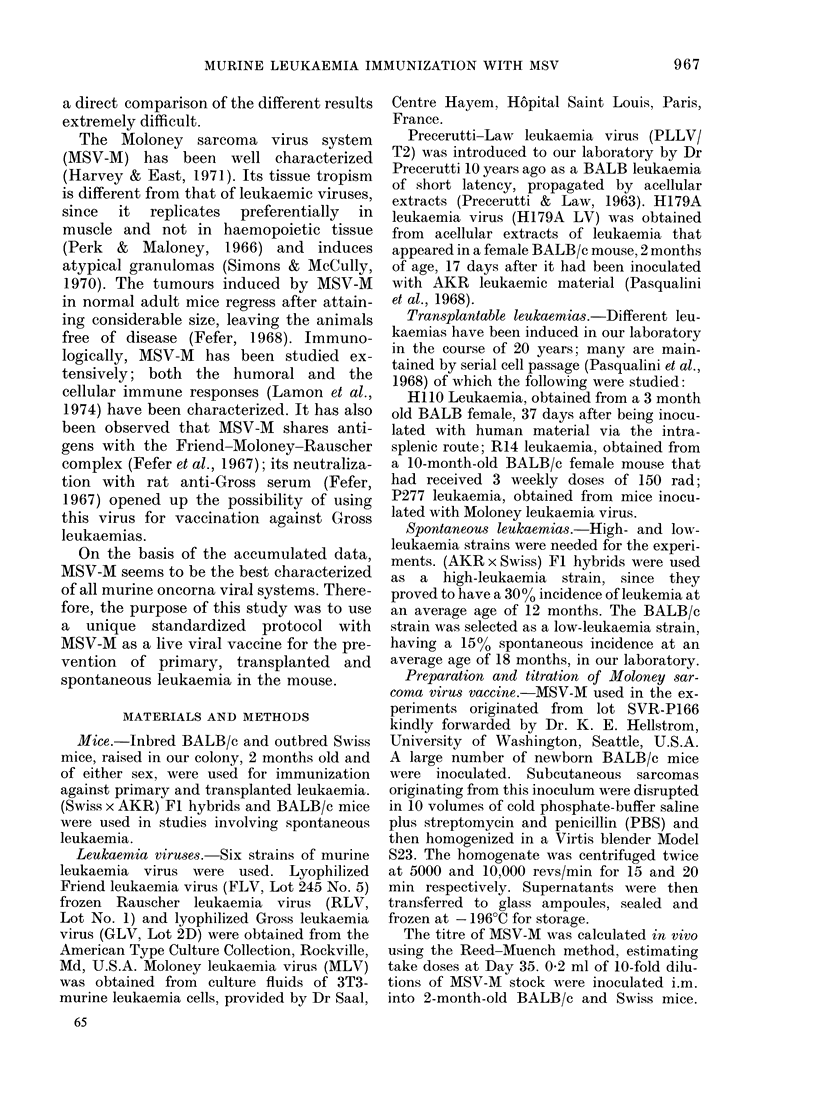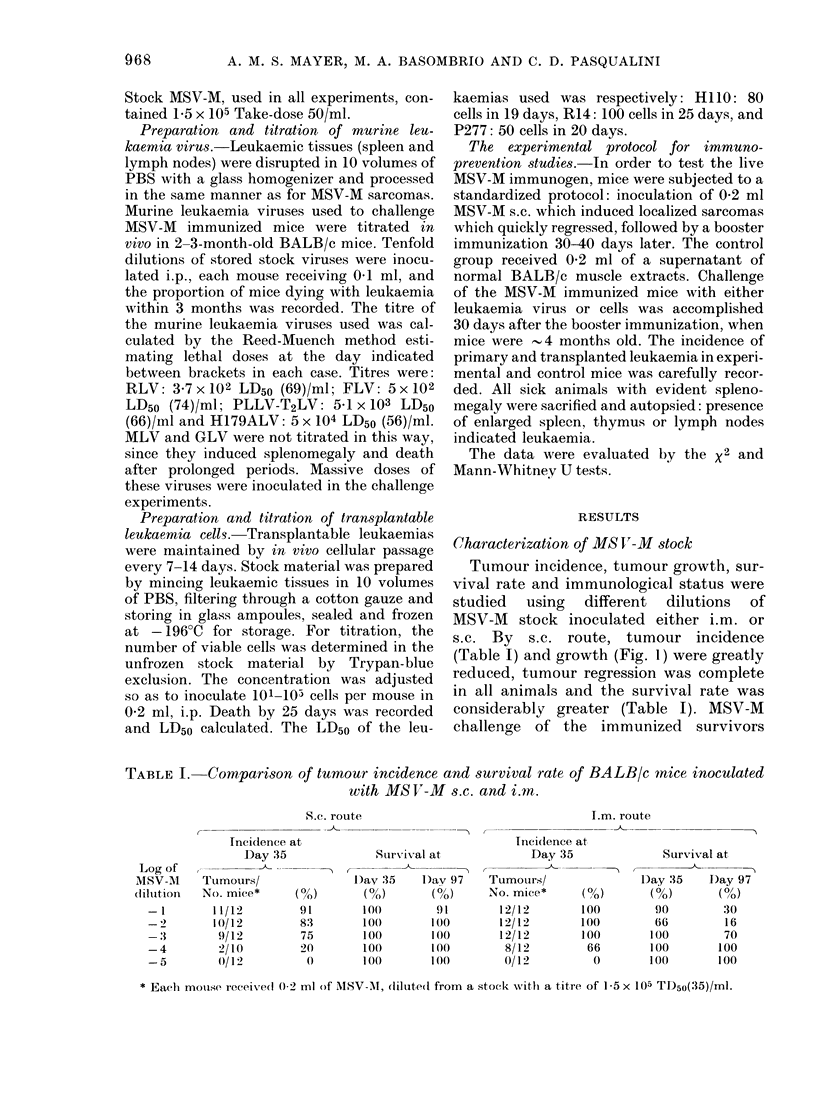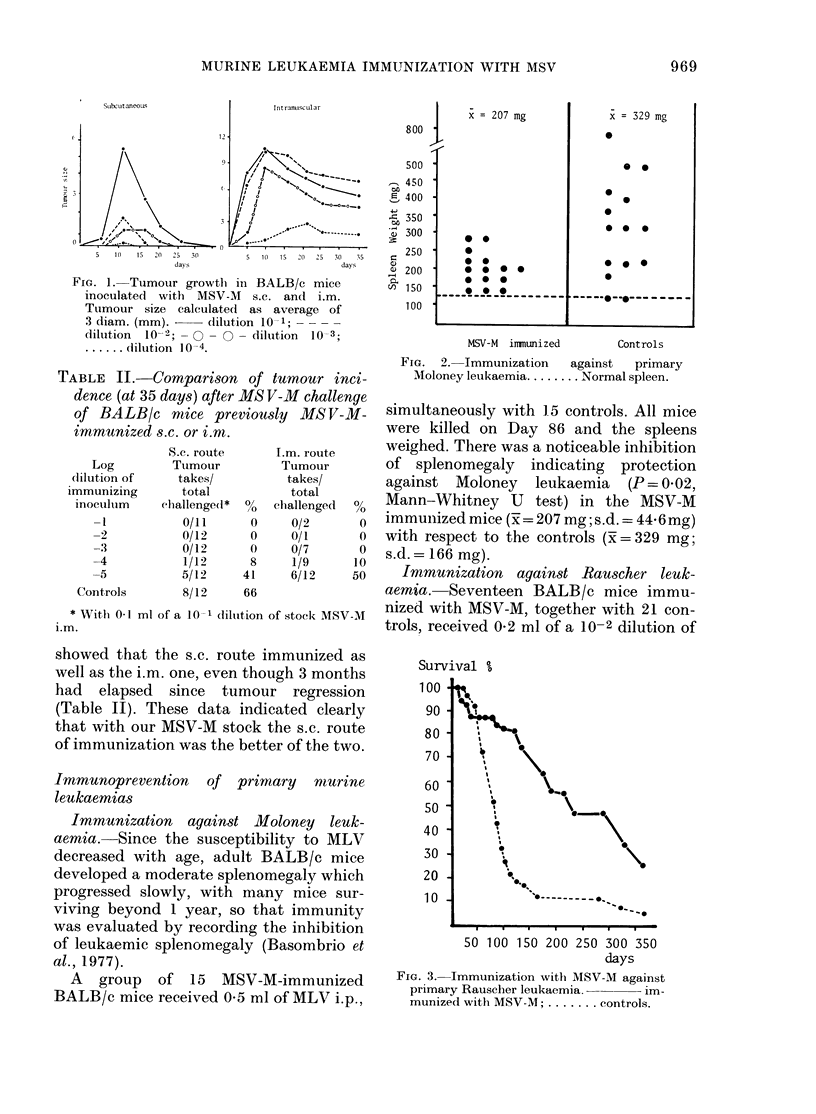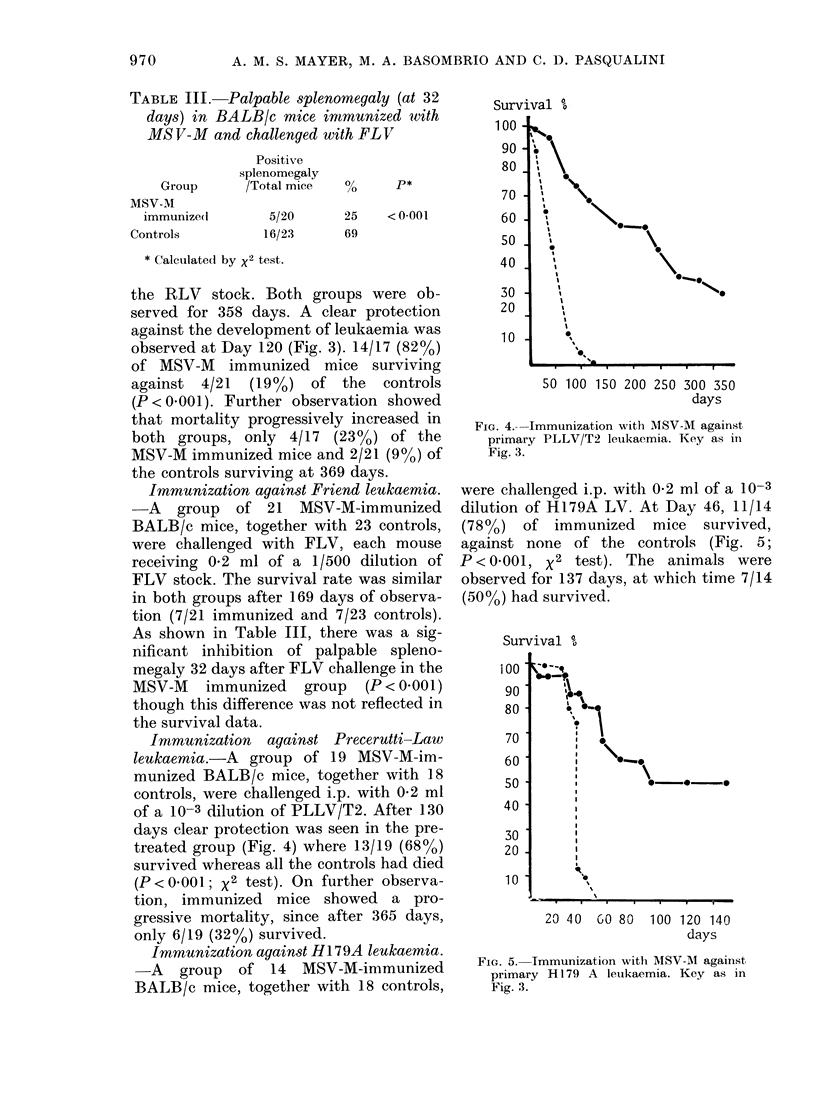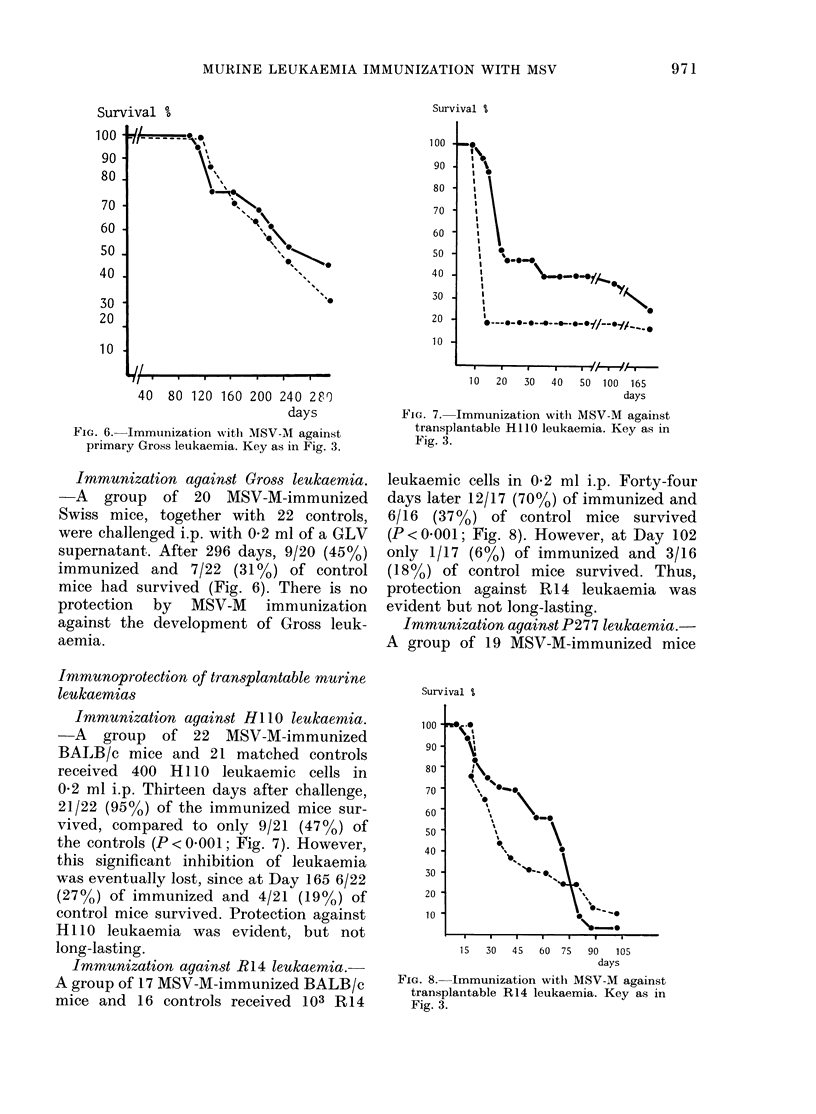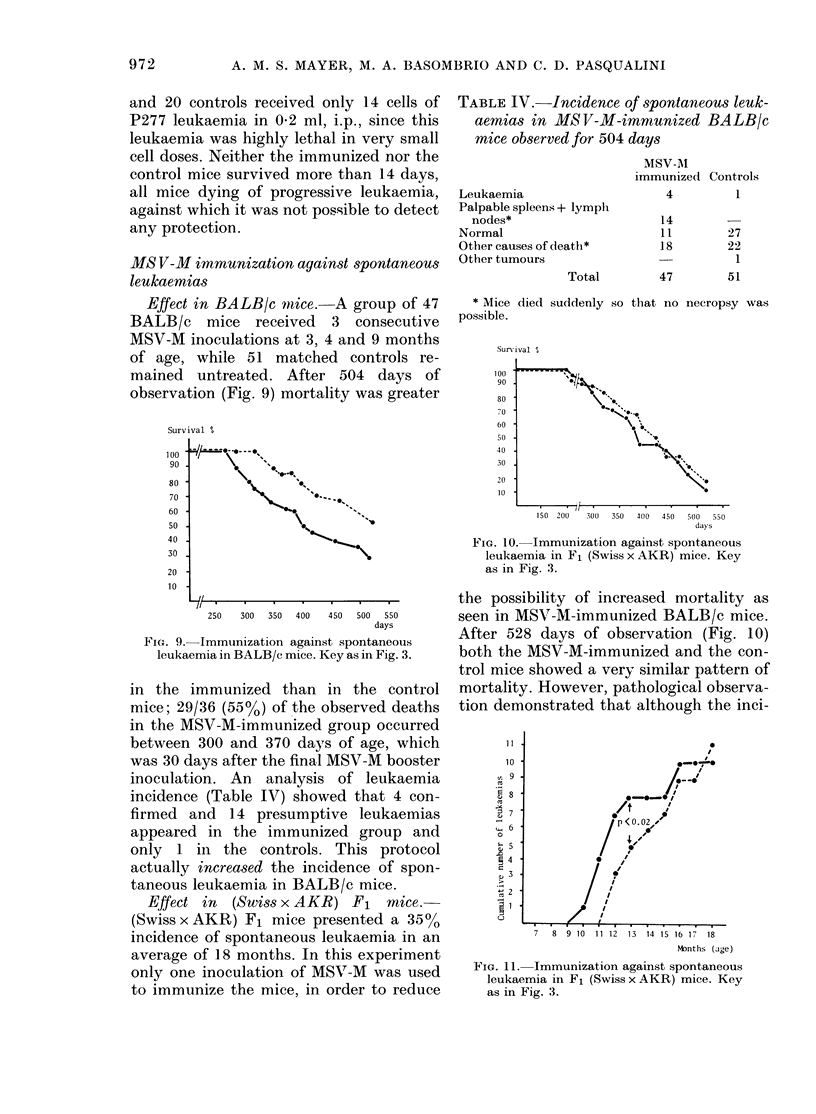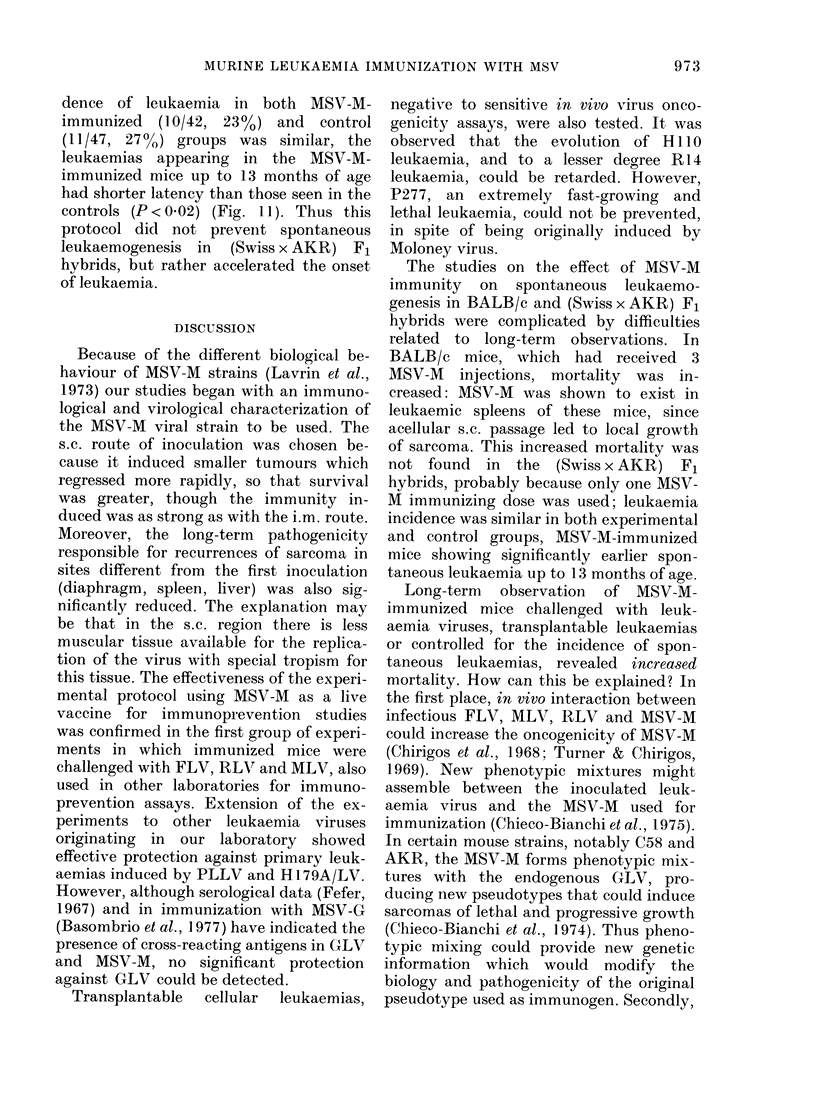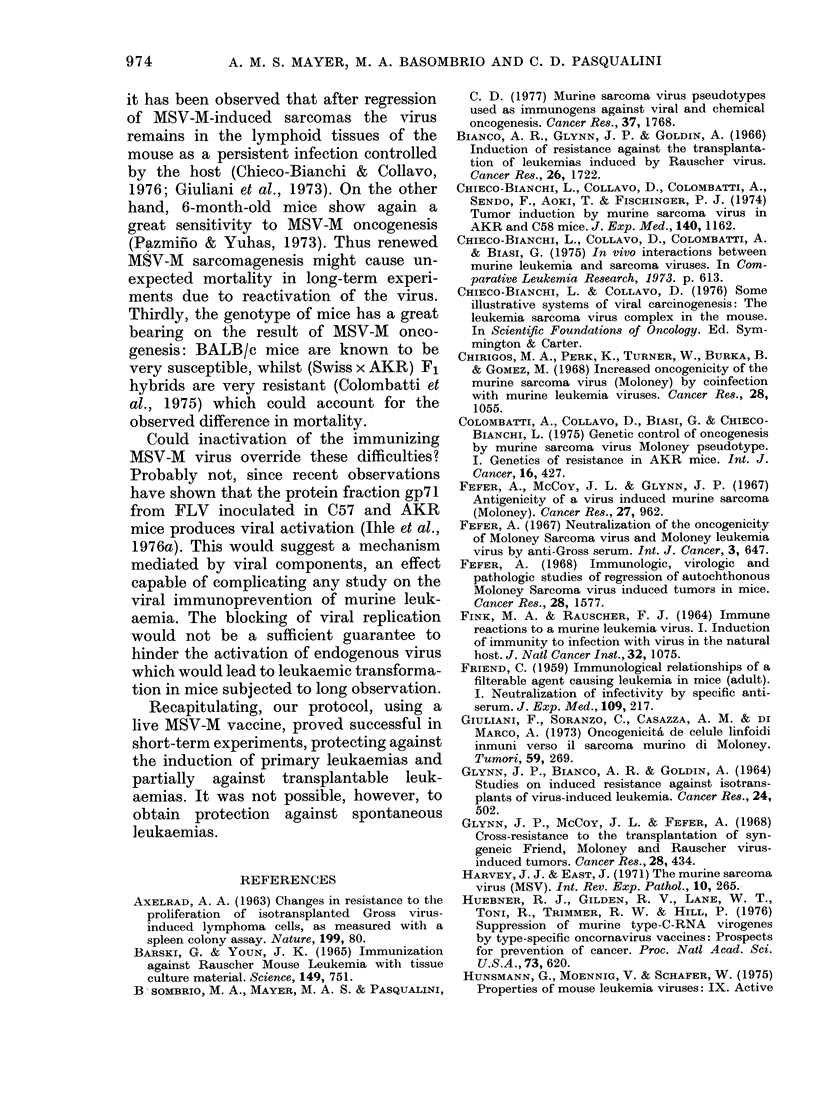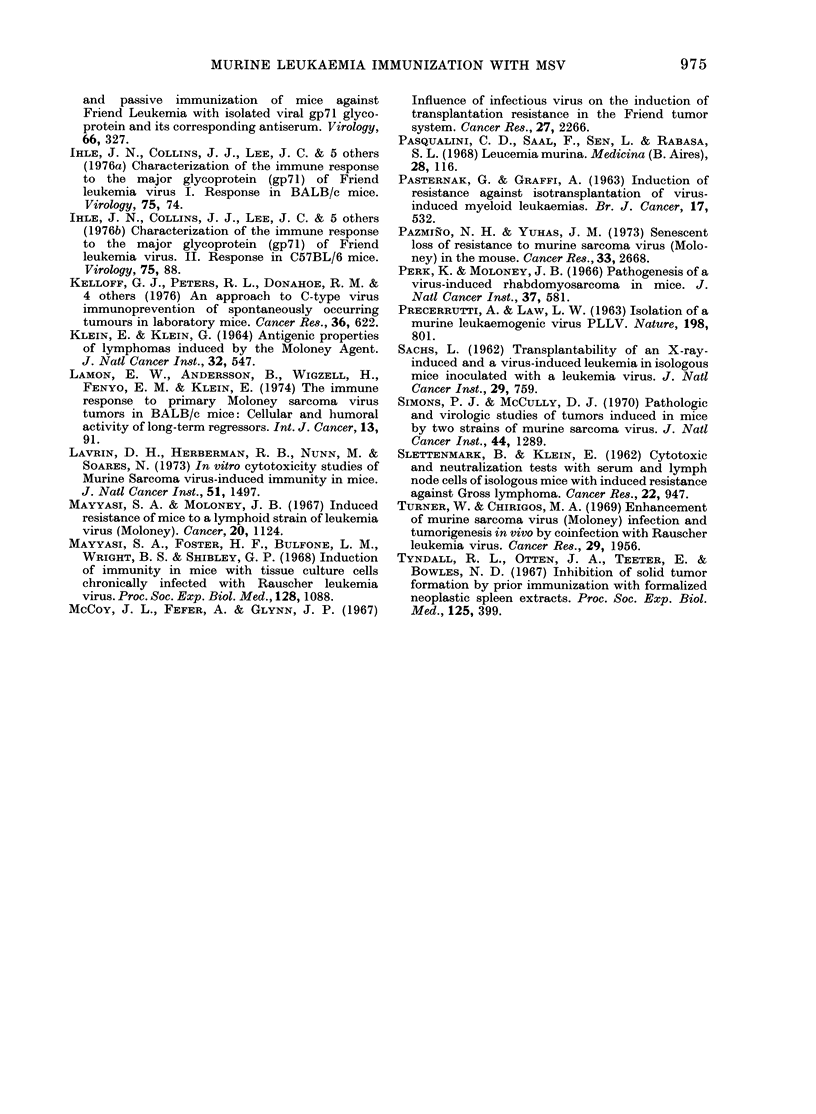# Immunization against primary, transplanted and spontaneous murine leukaemia using a live Moloney sarcoma virus vaccine.

**DOI:** 10.1038/bjc.1980.175

**Published:** 1980-06

**Authors:** A. M. Mayer, M. A. Basombrio, C. D. Pasqualini

## Abstract

The purpose of this study was to use an immunization protocol with Moloney sarcoma virus (MSV-M) as active immunogen against exogenous and endogenous leukaemia. The s.c. route was chosen since it offered advantages over the i.m. route: the primary sarcomas were smaller, the regression faster, there were fewer recurrences and there was good persistent immunity. Strong protection was obtained against primary leukaemias induced by Friend leukaemia virus (FLV), Moloney leukaemia virus (MLV), Rauscher leukaemia virus (RLV), Precerutti-Law leukaemia virus (PLLV/T2), and H179A leukaemia virus. It was not possible to protect against leukaemia induced by Gross leukaemia virus (GLV). With transplantable leukaemias the results varied: partial protection was observed against H110 leukaemia (induced with human material) and R14 leukaemia (induced by X-irradiation) whilst no protection was obtained against P277 leukaemia (induced by Moloney leukaemia virus). As for spontaneous leukaemias, immunized BALB/c mice showed an increased incidence over the controls, while in F1 (Swiss x AKR) mice the incidence was similar but the latent period was shorter. Furthermore, in long-term observations the MSV-M-immunized mice showed an increased mortaltiy, which could be related to (1) new phenotypic mixtures between MSV-M and leukaemia viruses; (2) reactivation of MSV-M sarcoma-genesis with age, and (3) genotype susceptibility to MSV-M.


*Br. J. Cancer* (1980) **41**, 966

### IMMUNIZATION AGAINST PRIMARY, TRANSPLANTED AND SPONTANEOUS MURINE LEUKAEMIA USING A LIVE MOLONEY SARCOMA VIRUS VACCINE

A. M. S. MAYER,* M. A. BASOMBRIO† AND C. D. PASQUALINI†

*From the Sección Leucemia Experimental, Instituto de Investigaciones Hematólogicas, Academia Nacional de Medicina, Las Heras* 3092, 1425, *Buenos Aires, Argentina*

Received 9 October 1979 Accepted 16 January 1980

**Summary.—The purpose of this study was to use an immunization protocol with Moloney sarcoma virus (MSV-M) as active immunogen against exogenous and endogenous leukaemia. The s.c. route was chosen since it offered advantages over the i.m. route: the primary sarcomas were smaller, the regression faster, there were fewer recurrences and there was good persistent immunity. Strong protection was obtained against primary leukaemias induced by Friend leukaemia virus (FLV), Moloney leukaemia virus (MLV), Rauscher leukaemia virus (RLV), Precerutti-Law leukaemia virus (PLLV/T2), and H179A leukaemia virus. It was not possible to protect against leukaemia induced by Gross leukaemia virus (GLV). With transplantable leukaemias the results varied: partial protection was observed against H110 leukaemia (induced with human material) and R14 leukaemia (induced by X-irradiation) whilst no protection was obtained against P277 leukaemia (induced by Moloney leukaemia virus).**

**As for spontaneous leukaemias, immunized BALB/c mice showed an increased incidence over the controls, while in F1 (Swiss × AKR) mice the incidence was similar but the latent period was shorter. Furthermore, in long-term observations the MSV-M-immunized mice showed an increased mortality, which could be related to (1) new phenotypic mixtures between MSV-M and leukaemia viruses; (2) reactivation of MSV-M sarcoma-genesis with age, and (3) genotype susceptibility to MSV-M.**

To DATE, no standardized system has been used to compare the results of one and the same immunization protocol on the prevention of transplanted, primary and spontaneous leukaemia in the mouse. However, on different occasions, active immunization methods have been used, such as: (1) syngeneic transplantable leukaemia in sub-threshold doses (Axelrad, 1963; Klein & Klein, 1964; Pasternak & Graffi, 1963); (2) allogeneic transplantable leukaemia (Klein & Klein, 1964; McCoy *et al.*, 1967; Pasternak & Graffi, 1963); (3) culture cells chronically infected with virus (Barski & Youn, 1965; Mayyasi *et al.*, 1968); (4) infectious leukaemia virus (Bianco *et al.*, 1966; Glynn *et al.*, 1964, 1968; Klein & Klein, 1964; Mayyasi & Moloney, 1967; McCoy *et al.*, 1967; Sachs, 1962; Slettenmark & Klein, 1962); (5) attenuated leukaemia virus (Fink & Rauscher, 1964; Friend, 1959; Huebner *et al.*, 1976; Kelloff *et al.*, 1976; Mayyasi & Moloney, 1967; McCoy *et al.*, 1967; Tyndall, *et al.*, 1967); (6) infectious murine sarcoma virus (Fefer *et al.*, 1967; Huebner *et al.*, 1976; Basombrio *et al.*, 1977); and (7) purified viral components (Hunsmann *et al.*, 1975; Ihle *et al.*, 1976*a,b*). The variability among experimental protocols makes

* By whom this work was submitted in partial fulfilment of the requirements for the degree of Doctor of Philosophy in Biological Sciences at the University of Buenos Aires, Buenos Aires, Argentina.

† Member of Research Career, CONICET (Consejo Nacional de Inves tigaciones Científicas y Técnicas).

Address for reprints: Christiane Dosne Pasqualini, Academia Nacional de Medicina, Las Heras 3092, 1425 Buenos Aires, Argentina.

a direct comparison of the different results extremely difficult.

The Moloney sarcoma virus system (MSV-M) has been well characterized (Harvey & East, 1971). Its tissue tropism is different from that of leukaemic viruses, since it replicates preferentially in muscle and not in haemopoietic tissue (Perk & Maloney, 1966) and induces atypical granulomas (Simons & McCully, 1970). The tumours induced by MSV-M in normal adult mice regress after attaining considerable size, leaving the animals free of disease (Fefer, 1968). Immunologically, MSV-M has been studied extensively; both the humoral and the cellular immune responses (Lamon *et al.*, 1974) have been characterized. It has also been observed that MSV-M shares antigens with the Friend–Moloney–Rauscher complex (Fefer *et al.*, 1967); its neutralization with rat anti-Gross serum (Fefer, 1967) opened up the possibility of using this virus for vaccination against Gross leukaemias.

On the basis of the accumulated data, MSV-M seems to be the best characterized of all murine oncorna viral systems. Therefore, the purpose of this study was to use a unique standardized protocol with MSV-M as a live viral vaccine for the prevention of primary, transplanted and spontaneous leukaemia in the mouse.

### MATERIALS AND METHODS

*Mice.*—Inbred BALB/c and outbred Swiss mice, raised in our colony, 2 months old and of either sex, were used for immunization against primary and transplanted leukaemia. (Swiss x AKR) F1 hybrids and BALB/c mice were used in studies involving spontaneous leukaemia.

*Leukaemia viruses.*—Six strains of murine leukaemia virus were used. Lyophilized Friend leukaemia virus (FLV, Lot 245 No. 5) frozen Rauscher leukaemia virus (RLV, Lot No. 1) and lyophilized Gross leukaemia virus (GLV, Lot 2D) were obtained from the American Type Culture Collection, Rockville, Md, U.S.A. Moloney leukaemia virus (MLV) was obtained from culture fluids of 3T3-murine leukaemia cells, provided by Dr Saal, Centre Hayem, Hôpital Saint Louis, Paris, France.

Precerutti–Law leukaemia virus (PLLV/T2) was introduced to our laboratory by Dr Precerutti 10 years ago as a BALB leukaemia of short latency, propagated by acellular extracts (Precerutti & Law, 1963). H179A leukaemia virus (H179A LV) was obtained from acellular extracts of leukaemia that appeared in a female BALB/c mouse, 2 months of age, 17 days after it had been inoculated with AKR leukaemic material (Pasqualini *et al.*, 1968).

*Transplantable leukaemias.*—Different leukaemias have been induced in our laboratory in the course of 20 years; many are maintained by serial cell passage (Pasqualini *et al.*, 1968) of which the following were studied:

H110 Leukaemia, obtained from a 3 month old BALB female, 37 days after being inoculated with human material via the intrasplenic route; R14 leukaemia, obtained from a 10-month-old BALB/c female mouse that had received 3 weekly doses of 150 rad; P277 leukaemia, obtained from mice inoculated with Moloney leukaemia virus.

*Spontaneous leukaemias.*—High- and low-leukaemia strains were needed for the experiments. (AKR x Swiss) F1 hybrids were used as a high-leukaemia strain, since they proved to have a 30% incidence of leukemia at an average age of 12 months. The BALB/c strain was selected as a low-leukaemia strain, having a 15% spontaneous incidence at an average age of 18 months, in our laboratory.

*Preparation and titration of Moloney sarcoma virus vaccine.*—MSV-M used in the experiments originated from lot SVR-P166 kindly forwarded by Dr. K. E. Hellstrom, University of Washington, Seattle, U.S.A. A large number of newborn BALB/c mice were inoculated. Subcutaneous sarcomas originating from this inoculum were disrupted in 10 volumes of cold phosphate-buffer saline plus streptomycin and penicillin (PBS) and then homogenized in a Virtis blender Model S23. The homogenate was centrifuged twice at 5000 and 10,000 revs/min for 15 and 20 min respectively. Supernatants were then transferred to glass ampoules, sealed and frozen at −196°C for storage.

The titre of MSV-M was calculated *in vivo* using the Reed–Muench method, estimating take doses at Day 35. 0·2 ml of 10-fold dilutions of MSV-M stock were inoculated i.m. into 2-month-old BALB/c and Swiss mice.

Stock MSV-M, used in all experiments, contained $1{\cdot}5 \times 10^5$ Take-dose 50/ml.

*Preparation and titration of murine leukaemia virus.*—Leukaemic tissues (spleen and lymph nodes) were disrupted in 10 volumes of PBS with a glass homogenizer and processed in the same manner as for MSV-M sarcomas. Murine leukaemia viruses used to challenge MSV-M immunized mice were titrated *in vivo* in 2–3-month-old BALB/c mice. Tenfold dilutions of stored stock viruses were inoculated i.p., each mouse receiving 0·1 ml, and the proportion of mice dying with leukaemia within 3 months was recorded. The titre of the murine leukaemia viruses used was calculated by the Reed-Muench method estimating lethal doses at the day indicated between brackets in each case. Titres were: RLV: $3{\cdot}7 \times 10^2$ $LD_{50}$ (69)/ml; FLV: $5 \times 10^2$ $LD_{50}$ (74)/ml; PLLV-$T_2$LV: $5{\cdot}1 \times 10^3$ $LD_{50}$ (66)/ml and H179ALV: $5 \times 10^4$ $LD_{50}$ (56)/ml. MLV and GLV were not titrated in this way, since they induced splenomegaly and death after prolonged periods. Massive doses of these viruses were inoculated in the challenge experiments.

*Preparation and titration of transplantable leukaemia cells.*—Transplantable leukaemias were maintained by *in vivo* cellular passage every 7–14 days. Stock material was prepared by mincing leukaemic tissues in 10 volumes of PBS, filtering through a cotton gauze and storing in glass ampoules, sealed and frozen at −196°C for storage. For titration, the number of viable cells was determined in the unfrozen stock material by Trypan-blue exclusion. The concentration was adjusted so as to inoculate $10^1$–$10^5$ cells per mouse in 0·2 ml, i.p. Death by 25 days was recorded and $LD_{50}$ calculated. The $LD_{50}$ of the leukaemias used was respectively: H110: 80 cells in 19 days, R14: 100 cells in 25 days, and P277: 50 cells in 20 days.

*The experimental protocol for immunoprevention studies.*—In order to test the live MSV-M immunogen, mice were subjected to a standardized protocol: inoculation of 0·2 ml MSV-M s.c. which induced localized sarcomas which quickly regressed, followed by a booster immunization 30–40 days later. The control group received 0·2 ml of a supernatant of normal BALB/c muscle extracts. Challenge of the MSV-M immunized mice with either leukaemia virus or cells was accomplished 30 days after the booster immunization, when mice were ~4 months old. The incidence of primary and transplanted leukaemia in experimental and control mice was carefully recorded. All sick animals with evident splenomegaly were sacrified and autopsied: presence of enlarged spleen, thymus or lymph nodes indicated leukaemia.

The data were evaluated by the $\chi^2$ and Mann-Whitney U tests.

### RESULTS

### *Characterization of MSV-M stock*

Tumour incidence, tumour growth, survival rate and immunological status were studied using different dilutions of MSV-M stock inoculated either i.m. or s.c. By s.c. route, tumour incidence (Table I) and growth (Fig. 1) were greatly reduced, tumour regression was complete in all animals and the survival rate was considerably greater (Table I). MSV-M challenge of the immunized survivors

TABLE I.—*Comparison of tumour incidence and survival rate of BALB/c mice inoculated with MSV-M s.c. and i.m.*

| | S.c. route | | | | I.m. route | | | |
|---|---|---|---|---|---|---|---|---|
| | Incidence at Day 35 | | Survival at | | Incidence at Day 35 | | Survival at | |
| Log of MSV-M dilution | Tumours/ No. mice* | (%) | Day 35 (%) | Day 97 (%) | Tumours/ No. mice* | (%) | Day 35 (%) | Day 97 (%) |
| −1 | 11/12 | 91 | 100 | 91 | 12/12 | 100 | 90 | 30 |
| −2 | 10/12 | 83 | 100 | 100 | 12/12 | 100 | 66 | 16 |
| −3 | 9/12 | 75 | 100 | 100 | 12/12 | 100 | 100 | 70 |
| −4 | 2/10 | 20 | 100 | 100 | 8/12 | 66 | 100 | 100 |
| −5 | 0/12 | 0 | 100 | 100 | 0/12 | 0 | 100 | 100 |

* Each mouse received 0·2 ml of MSV-M, diluted from a stock with a titre of $1{\cdot}5 \times 10^5$ $TD_{50}$(35)/ml.

FIG. 1.—Tumour growth in BALB/c mice inoculated with MSV-M s.c. and i.m. Tumour size calculated as average of 3 diam. (mm). —— dilution $10^{-1}$; – – – – dilution $10^{-2}$; – ○ – ○ – dilution $10^{-3}$; ...... dilution $10^{-4}$.

TABLE II.—*Comparison of tumour incidence (at 35 days) after MSV-M challenge of BALB/c mice previously MSV-M-immunized s.c. or i.m.*

| Log dilution of immunizing inoculum | S.c. route Tumour takes/ total challenged* | % | I.m. route Tumour takes/ total challenged | % |
|---|---|---|---|---|
| -1 | 0/11 | 0 | 0/2 | 0 |
| -2 | 0/12 | 0 | 0/1 | 0 |
| -3 | 0/12 | 0 | 0/7 | 0 |
| -4 | 1/12 | 8 | 1/9 | 10 |
| -5 | 5/12 | 41 | 6/12 | 50 |
| Controls | 8/12 | 66 | | |

* With 0·1 ml of a $10^{-1}$ dilution of stock MSV-M i.m.

showed that the s.c. route immunized as well as the i.m. one, even though 3 months had elapsed since tumour regression (Table II). These data indicated clearly that with our MSV-M stock the s.c. route of immunization was the better of the two.

*Immunoprevention of primary murine leukaemias*

*Immunization against Moloney leukaemia.*—Since the susceptibility to MLV decreased with age, adult BALB/c mice developed a moderate splenomegaly which progressed slowly, with many mice surviving beyond 1 year, so that immunity was evaluated by recording the inhibition of leukaemic splenomegaly (Basombrio *et al.*, 1977).

A group of 15 MSV-M-immunized BALB/c mice received 0·5 ml of MLV i.p., simultaneously with 15 controls. All mice were killed on Day 86 and the spleens weighed. There was a noticeable inhibition of splenomegaly indicating protection against Moloney leukaemia ($P = 0{\cdot}02$, Mann–Whitney U test) in the MSV-M immunized mice ($\bar{x} = 207$ mg; s.d. = 44·6 mg) with respect to the controls ($\bar{x} = 329$ mg; s.d. = 166 mg).

FIG. 2.—Immunization against primary Moloney leukaemia. . . . . . . . Normal spleen.

*Immunization against Rauscher leukaemia.*—Seventeen BALB/c mice immunized with MSV-M, together with 21 controls, received 0·2 ml of a $10^{-2}$ dilution of

FIG. 3.—Immunization with MSV-M against primary Rauscher leukaemia. ———— immunized with MSV-M; . . . . . . . controls.

TABLE III.—*Palpable splenomegaly (at 32 days) in BALB/c mice immunized with MSV-M and challenged with FLV*

| Group | Positive splenomegaly /Total mice | % | P* |
|---|---|---|---|
| MSV-M immunized | 5/20 | 25 | < 0·001 |
| Controls | 16/23 | 69 | |

* Calculated by $\chi^2$ test.

the RLV stock. Both groups were observed for 358 days. A clear protection against the development of leukaemia was observed at Day 120 (Fig. 3). 14/17 (82%) of MSV-M immunized mice surviving against 4/21 (19%) of the controls ($P < 0·001$). Further observation showed that mortality progressively increased in both groups, only 4/17 (23%) of the MSV-M immunized mice and 2/21 (9%) of the controls surviving at 369 days.

*Immunization against Friend leukaemia.*—A group of 21 MSV-M-immunized BALB/c mice, together with 23 controls, were challenged with FLV, each mouse receiving 0·2 ml of a 1/500 dilution of FLV stock. The survival rate was similar in both groups after 169 days of observation (7/21 immunized and 7/23 controls). As shown in Table III, there was a significant inhibition of palpable splenomegaly 32 days after FLV challenge in the MSV-M immunized group ($P < 0·001$) though this difference was not reflected in the survival data.

*Immunization against Precerutti–Law leukaemia.*—A group of 19 MSV-M-immunized BALB/c mice, together with 18 controls, were challenged i.p. with 0·2 ml of a $10^{-3}$ dilution of PLLV/T2. After 130 days clear protection was seen in the pretreated group (Fig. 4) where 13/19 (68%) survived whereas all the controls had died ($P < 0·001$; $\chi^2$ test). On further observation, immunized mice showed a progressive mortality, since after 365 days, only 6/19 (32%) survived.

*Immunization against H179A leukaemia.*—A group of 14 MSV-M-immunized BALB/c mice, together with 18 controls, were challenged i.p. with 0·2 ml of a $10^{-3}$ dilution of H179A LV. At Day 46, 11/14 (78%) of immunized mice survived, against none of the controls (Fig. 5; $P < 0·001$, $\chi^2$ test). The animals were observed for 137 days, at which time 7/14 (50%) had survived.

FIG. 4.—Immunization with MSV-M against primary PLLV/T2 leukaemia. Key as in Fig. 3.

FIG. 5.—Immunization with MSV-M against primary H179 A leukaemia. Key as in Fig. 3.

FIG. 6.—Immunization with MSV-M against primary Gross leukaemia. Key as in Fig. 3.

FIG. 7.—Immunization with MSV-M against transplantable H110 leukaemia. Key as in Fig. 3.

*Immunization against Gross leukaemia.*—A group of 20 MSV-M-immunized Swiss mice, together with 22 controls, were challenged i.p. with 0·2 ml of a GLV supernatant. After 296 days, 9/20 (45%) immunized and 7/22 (31%) of control mice had survived (Fig. 6). There is no protection by MSV-M immunization against the development of Gross leukaemia.

*Immunoprotection of transplantable murine leukaemias*

*Immunization against H110 leukaemia.*—A group of 22 MSV-M-immunized BALB/c mice and 21 matched controls received 400 H110 leukaemic cells in 0·2 ml i.p. Thirteen days after challenge, 21/22 (95%) of the immunized mice survived, compared to only 9/21 (47%) of the controls ($P < 0{\cdot}001$; Fig. 7). However, this significant inhibition of leukaemia was eventually lost, since at Day 165 6/22 (27%) of immunized and 4/21 (19%) of control mice survived. Protection against H110 leukaemia was evident, but not long-lasting.

*Immunization against R14 leukaemia.*—A group of 17 MSV-M-immunized BALB/c mice and 16 controls received $10^3$ R14 leukaemic cells in 0·2 ml i.p. Forty-four days later 12/17 (70%) of immunized and 6/16 (37%) of control mice survived ($P < 0{\cdot}001$; Fig. 8). However, at Day 102 only 1/17 (6%) of immunized and 3/16 (18%) of control mice survived. Thus, protection against R14 leukaemia was evident but not long-lasting.

*Immunization against P277 leukaemia.*—A group of 19 MSV-M-immunized mice

FIG. 8.—Immunization with MSV-M against transplantable R14 leukaemia. Key as in Fig. 3.

and 20 controls received only 14 cells of P277 leukaemia in 0·2 ml, i.p., since this leukaemia was highly lethal in very small cell doses. Neither the immunized nor the control mice survived more than 14 days, all mice dying of progressive leukaemia, against which it was not possible to detect any protection.

*MSV-M immunization against spontaneous leukaemias*

*Effect in BALB/c mice.*—A group of 47 BALB/c mice received 3 consecutive MSV-M inoculations at 3, 4 and 9 months of age, while 51 matched controls remained untreated. After 504 days of observation (Fig. 9) mortality was greater

FIG. 9.—Immunization against spontaneous leukaemia in BALB/c mice. Key as in Fig. 3.

in the immunized than in the control mice; 29/36 (55%) of the observed deaths in the MSV-M-immunized group occurred between 300 and 370 days of age, which was 30 days after the final MSV-M booster inoculation. An analysis of leukaemia incidence (Table IV) showed that 4 confirmed and 14 presumptive leukaemias appeared in the immunized group and only 1 in the controls. This protocol actually *increased* the incidence of spontaneous leukaemia in BALB/c mice.

*Effect in (Swiss × AKR) $F_1$ mice.*—(Swiss × AKR) $F_1$ mice presented a 35% incidence of spontaneous leukaemia in an average of 18 months. In this experiment only one inoculation of MSV-M was used to immunize the mice, in order to reduce

TABLE IV.—*Incidence of spontaneous leukaemias in MSV-M-immunized BALB/c mice observed for* 504 *days*

| | MSV-M immunized | Controls |
|---|---|---|
| Leukaemia | 4 | 1 |
| Palpable spleens + lymph nodes* | 14 | — |
| Normal | 11 | 27 |
| Other causes of death* | 18 | 22 |
| Other tumours | — | 1 |
| Total | 47 | 51 |

* Mice died suddenly so that no necropsy was possible.

FIG. 10.—Immunization against spontaneous leukaemia in $F_1$ (Swiss × AKR) mice. Key as in Fig. 3.

the possibility of increased mortality as seen in MSV-M-immunized BALB/c mice. After 528 days of observation (Fig. 10) both the MSV-M-immunized and the control mice showed a very similar pattern of mortality. However, pathological observation demonstrated that although the inci-

FIG. 11.—Immunization against spontaneous leukaemia in $F_1$ (Swiss × AKR) mice. Key as in Fig. 3.

dence of leukaemia in both MSV-M-immunized (10/42, 23%) and control (11/47, 27%) groups was similar, the leukaemias appearing in the MSV-M-immunized mice up to 13 months of age had shorter latency than those seen in the controls ($P < 0.02$) (Fig. 11). Thus this protocol did not prevent spontaneous leukaemogenesis in (Swiss × AKR) $F_1$ hybrids, but rather accelerated the onset of leukaemia.

### DISCUSSION

Because of the different biological behaviour of MSV-M strains (Lavrin *et al.*, 1973) our studies began with an immunological and virological characterization of the MSV-M viral strain to be used. The s.c. route of inoculation was chosen because it induced smaller tumours which regressed more rapidly, so that survival was greater, though the immunity induced was as strong as with the i.m. route. Moreover, the long-term pathogenicity responsible for recurrences of sarcoma in sites different from the first inoculation (diaphragm, spleen, liver) was also significantly reduced. The explanation may be that in the s.c. region there is less muscular tissue available for the replication of the virus with special tropism for this tissue. The effectiveness of the experimental protocol using MSV-M as a live vaccine for immunoprevention studies was confirmed in the first group of experiments in which immunized mice were challenged with FLV, RLV and MLV, also used in other laboratories for immunoprevention assays. Extension of the experiments to other leukaemia viruses originating in our laboratory showed effective protection against primary leukaemias induced by PLLV and H179A/LV. However, although serological data (Fefer, 1967) and in immunization with MSV-G (Basombrio *et al.*, 1977) have indicated the presence of cross-reacting antigens in GLV and MSV-M, no significant protection against GLV could be detected.

Transplantable cellular leukaemias, negative to sensitive *in vivo* virus oncogenicity assays, were also tested. It was observed that the evolution of H110 leukaemia, and to a lesser degree R14 leukaemia, could be retarded. However, P277, an extremely fast-growing and lethal leukaemia, could not be prevented, in spite of being originally induced by Moloney virus.

The studies on the effect of MSV-M immunity on spontaneous leukaemogenesis in BALB/c and (Swiss × AKR) $F_1$ hybrids were complicated by difficulties related to long-term observations. In BALB/c mice, which had received 3 MSV-M injections, mortality was increased: MSV-M was shown to exist in leukaemic spleens of these mice, since acellular s.c. passage led to local growth of sarcoma. This increased mortality was not found in the (Swiss × AKR) $F_1$ hybrids, probably because only one MSV-M immunizing dose was used; leukaemia incidence was similar in both experimental and control groups, MSV-M-immunized mice showing significantly earlier spontaneous leukaemia up to 13 months of age.

Long-term observation of MSV-M-immunized mice challenged with leukaemia viruses, transplantable leukaemias or controlled for the incidence of spontaneous leukaemias, revealed *increased* mortality. How can this be explained? In the first place, *in vivo* interaction between infectious FLV, MLV, RLV and MSV-M could increase the oncogenicity of MSV-M (Chirigos *et al.*, 1968; Turner & Chirigos, 1969). New phenotypic mixtures might assemble between the inoculated leukaemia virus and the MSV-M used for immunization (Chieco-Bianchi *et al.*, 1975). In certain mouse strains, notably C58 and AKR, the MSV-M forms phenotypic mixtures with the endogenous GLV, producing new pseudotypes that could induce sarcomas of lethal and progressive growth (Chieco-Bianchi *et al.*, 1974). Thus phenotypic mixing could provide new genetic information which would modify the biology and pathogenicity of the original pseudotype used as immunogen. Secondly, it has been observed that after regression of MSV-M-induced sarcomas the virus remains in the lymphoid tissues of the mouse as a persistent infection controlled by the host (Chieco-Bianchi & Collavo, 1976; Giuliani *et al.*, 1973). On the other hand, 6-month-old mice show again a great sensitivity to MSV-M oncogenesis (Pazmiño & Yuhas, 1973). Thus renewed MSV-M sarcomagenesis might cause unexpected mortality in long-term experiments due to reactivation of the virus. Thirdly, the genotype of mice has a great bearing on the result of MSV-M oncogenesis: BALB/c mice are known to be very susceptible, whilst (Swiss × AKR) $F_1$ hybrids are very resistant (Colombatti *et al.*, 1975) which could account for the observed difference in mortality.

Could inactivation of the immunizing MSV-M virus override these difficulties? Probably not, since recent observations have shown that the protein fraction gp71 from FLV inoculated in C57 and AKR mice produces viral activation (Ihle *et al.*, 1976*a*). This would suggest a mechanism mediated by viral components, an effect capable of complicating any study on the viral immunoprevention of murine leukaemia. The blocking of viral replication would not be a sufficient guarantee to hinder the activation of endogenous virus which would lead to leukaemic transformation in mice subjected to long observation.

Recapitulating, our protocol, using a live MSV-M vaccine, proved successful in short-term experiments, protecting against the induction of primary leukaemias and partially against transplantable leukaemias. It was not possible, however, to obtain protection against spontaneous leukaemias.

### REFERENCES

Axelrad, A. A. (1963) Changes in resistance to the proliferation of isotransplanted Gross virus-induced lymphoma cells, as measured with a spleen colony assay. *Nature*, **199**, 80.

Barski, G. & Youn, J. K. (1965) Immunization against Rauscher Mouse Leukemia with tissue culture material. *Science*, **149**, 751.

B[illegible]sombrio, M. A., Mayer, M. A. S. & Pasqualini, C. D. (1977) Murine sarcoma virus pseudotypes used as immunogens against viral and chemical oncogenesis. *Cancer Res.*, **37**, 1768.

Bianco, A. R., Glynn, J. P. & Goldin, A. (1966) Induction of resistance against the transplantation of leukemias induced by Rauscher virus. *Cancer Res.*, **26**, 1722.

Chieco-Bianchi, L., Collavo, D., Colombatti, A., Sendo, F., Aoki, T. & Fischinger, P. J. (1974) Tumor induction by murine sarcoma virus in AKR and C58 mice. *J. Exp. Med.*, **140**, 1162.

Chieco-Bianchi, L., Collavo, D., Colombatti, A. & Biasi, G. (1975) *In vivo* interactions between murine leukemia and sarcoma viruses. In *Comparative Leukemia Research, 1973*. p. 613.

Chieco-Bianchi, L. & Collavo, D. (1976) Some illustrative systems of viral carcinogenesis: The leukemia sarcoma virus complex in the mouse. In *Scientific Foundations of Oncology*. Ed. Symmington & Carter.

Chirigos, M. A., Perk, K., Turner, W., Burka, B. & Gomez, M. (1968) Increased oncogenicity of the murine sarcoma virus (Moloney) by coinfection with murine leukemia viruses. *Cancer Res.*, **28**, 1055.

Colombatti, A., Collavo, D., Biasi, G. & Chieco-Bianchi, L. (1975) Genetic control of oncogenesis by murine sarcoma virus Moloney pseudotype. I. Genetics of resistance in AKR mice. *Int. J. Cancer*, **16**, 427.

Fefer, A., McCoy, J. L. & Glynn, J. P. (1967) Antigenicity of a virus induced murine sarcoma (Moloney). *Cancer Res.*, **27**, 962.

Fefer, A. (1967) Neutralization of the oncogenicity of Moloney Sarcoma virus and Moloney leukemia virus by anti-Gross serum. *Int. J. Cancer*, **3**, 647.

Fefer, A. (1968) Immunologic, virologic and pathologic studies of regression of autochthonous Moloney Sarcoma virus induced tumors in mice. *Cancer Res.*, **28**, 1577.

Fink, M. A. & Rauscher, F. J. (1964) Immune reactions to a murine leukemia virus. I. Induction of immunity to infection with virus in the natural host. *J. Natl Cancer Inst.*, **32**, 1075.

Friend, C. (1959) Immunological relationships of a filterable agent causing leukemia in mice (adult). I. Neutralization of infectivity by specific antiserum. *J. Exp. Med.*, **109**, 217.

Giuliani, F., Soranzo, C., Casazza, A. M. & di Marco, A. (1973) Oncogenicitá de celule linfoidi inmuni verso il sarcoma murino di Moloney. *Tumori*, **59**, 269.

Glynn, J. P., Bianco, A. R. & Goldin, A. (1964) Studies on induced resistance against isotransplants of virus-induced leukemia. *Cancer Res.*, **24**, 502.

Glynn, J. P., McCoy, J. L. & Fefer, A. (1968) Cross-resistance to the transplantation of syngeneic Friend, Moloney and Rauscher virus-induced tumors. *Cancer Res.*, **28**, 434.

Harvey, J. J. & East, J. (1971) The murine sarcoma virus (MSV). *Int. Rev. Exp. Pathol.*, **10**, 265.

Huebner, R. J., Gilden, R. V., Lane, W. T., Toni, R., Trimmer, R. W. & Hill, P. (1976) Suppression of murine type-C-RNA virogenes by type-specific oncornavirus vaccines: Prospects for prevention of cancer. *Proc. Natl Acad. Sci. U.S.A.*, **73**, 620.

Hunsmann, G., Moennig, V. & Schafer, W. (1975) Properties of mouse leukemia viruses: IX. Active and passive immunization of mice against Friend Leukemia with isolated viral gp71 glycoprotein and its corresponding antiserum. *Virology*, **66**, 327.

IHLE, J. N., COLLINS, J. J., LEE, J. C. & 5 others (1976*a*) Characterization of the immune response to the major glycoprotein (gp71) of Friend leukemia virus I. Response in BALB/c mice. *Virology*, **75**, 74.

IHLE, J. N., COLLINS, J. J., LEE, J. C. & 5 others (1976*b*) Characterization of the immune response to the major glycoprotein (gp71) of Friend leukemia virus. II. Response in C57BL/6 mice. *Virology*, **75**, 88.

KELLOFF, G. J., PETERS, R. L., DONAHOE, R. M. & 4 others (1976) An approach to C-type virus immunoprevention of spontaneously occurring tumours in laboratory mice. *Cancer Res.*, **36**, 622.

KLEIN, E. & KLEIN, G. (1964) Antigenic properties of lymphomas induced by the Moloney Agent. *J. Natl Cancer Inst.*, **32**, 547.

LAMON, E. W., ANDERSSON, B., WIGZELL, H., FENYO, E. M. & KLEIN, E. (1974) The immune response to primary Moloney sarcoma virus tumors in BALB/c mice: Cellular and humoral activity of long-term regressors. *Int. J. Cancer*, **13**, 91.

LAVRIN, D. H., HERBERMAN, R. B., NUNN, M. & SOARES, N. (1973) *In vitro* cytotoxicity studies of Murine Sarcoma virus-induced immunity in mice. *J. Natl Cancer Inst.*, **51**, 1497.

MAYYASI, S. A. & MOLONEY, J. B. (1967) Induced resistance of mice to a lymphoid strain of leukemia virus (Moloney). *Cancer*, **20**, 1124.

MAYYASI, S. A., FOSTER, H. F., BULFONE, L. M., WRIGHT, B. S. & SHIBLEY, G. P. (1968) Induction of immunity in mice with tissue culture cells chronically infected with Rauscher leukemia virus. *Proc. Soc. Exp. Biol. Med.*, **128**, 1088.

MCCOY, J. L., FEFER, A. & GLYNN, J. P. (1967) Influence of infectious virus on the induction of transplantation resistance in the Friend tumor system. *Cancer Res.*, **27**, 2266.

PASQUALINI, C. D., SAAL, F., SEN, L. & RABASA, S. L. (1968) Leucemia murina. *Medicina* (B. Aires), **28**, 116.

PASTERNAK, G. & GRAFFI, A. (1963) Induction of resistance against isotransplantation of virus-induced myeloid leukaemias. *Br. J. Cancer*, **17**, 532.

PAZMIÑO, N. H. & YUHAS, J. M. (1973) Senescent loss of resistance to murine sarcoma virus (Moloney) in the mouse. *Cancer Res.*, **33**, 2668.

PERK, K. & MOLONEY, J. B. (1966) Pathogenesis of a virus-induced rhabdomyosarcoma in mice. *J. Natl Cancer Inst.*, **37**, 581.

PRECERRUTTI, A. & LAW, L. W. (1963) Isolation of a murine leukaemogenic virus PLLV. *Nature*, **198**, 801.

SACHS, L. (1962) Transplantability of an X-ray-induced and a virus-induced leukemia in isologous mice inoculated with a leukemia virus. *J. Natl Cancer Inst.*, **29**, 759.

SIMONS, P. J. & MCCULLY, D. J. (1970) Pathologic and virologic studies of tumors induced in mice by two strains of murine sarcoma virus. *J. Natl Cancer Inst.*, **44**, 1289.

SLETTENMARK, B. & KLEIN, E. (1962) Cytotoxic and neutralization tests with serum and lymph node cells of isologous mice with induced resistance against Gross lymphoma. *Cancer Res.*, **22**, 947.

TURNER, W. & CHIRIGOS, M. A. (1969) Enhancement of murine sarcoma virus (Moloney) infection and tumorigenesis *in vivo* by coinfection with Rauscher leukemia virus. *Cancer Res.*, **29**, 1956.

TYNDALL, R. L., OTTEN, J. A., TEETER, E. & BOWLES, N. D. (1967) Inhibition of solid tumor formation by prior immunization with formalized neoplastic spleen extracts. *Proc. Soc. Exp. Biol. Med.*, **125**, 399.